# Heme, Heme Oxygenase, and Endoplasmic Reticulum Stress—A New Insight into the Pathophysiology of Vascular Diseases

**DOI:** 10.3390/ijms20153675

**Published:** 2019-07-26

**Authors:** Tamás Gáll, György Balla, József Balla

**Affiliations:** 1 Department of Pediatrics, Faculty of Medicine, University of Debrecen, 4032 Debrecen, Hungary; 2 HAS-UD Vascular Biology and Myocardial Pathophysiology Research Group, Hungarian Academy of Sciences, 4032 Debrecen, Hungary; 3 Department of Internal Medicine, Faculty of Medicine, University of Debrecen, 4032 Debrecen, Hungary

**Keywords:** heme oxygenase, endoplasmic reticulum stress, hemoglobin, heme

## Abstract

The prevalence of vascular disorders continues to rise worldwide. Parallel with that, new pathophysiological pathways have been discovered, providing possible remedies for prevention and therapy in vascular diseases. Growing evidence suggests that endoplasmic reticulum (ER) stress is involved in a number of vasculopathies, including atherosclerosis, vascular brain events, and diabetes. Heme, which is released from hemoglobin or other heme proteins, triggers various pathophysiological consequence, including heme stress as well as ER stress. The potentially toxic free heme is converted by heme oxygenases (HOs) into carbon monoxide (CO), iron, and biliverdin (BV), the latter of which is reduced to bilirubin (BR). Redox-active iron is oxidized and stored by ferritin, an iron sequestering protein which exhibits ferroxidase activity. In recent years, CO, BV, and BR have been shown to control cellular processes such as inflammation, apoptosis, and antioxidant defense. This review covers our current knowledge about how heme induced endoplasmic reticulum stress (HIERS) participates in the pathogenesis of vascular disorders and highlights recent discoveries in the molecular mechanisms of HO-mediated cytoprotection in heme stress and ER stress, as well as crosstalk between ER stress and HO-1. Furthermore, we focus on the translational potential of HIERS and heme oxygenase-1 (HO-1) in atherosclerosis, diabetes mellitus, and brain hemorrhage.

## 1. Introduction

Hemoglobin (Hb) is not an innocent bystander of the pathophysiology in a number of diseases with extra- or intravascular hemorrhage/hemolysis. Free Hb, outside of red blood cells (RBCs), undergoes rapid oxidation from ferrous Hb (Fe^2+^) to metHb (Fe^3+^) that can be further oxidized to ferrylHb (Fe^4+=O2−^). Hb oxidation is followed by rapid heme release, resulting in free labile heme pools in the extracellular spaces [1]. Organ/tissue injuries, inherited hemolytic syndromes, sepsis, surgical interventions, brain hemorrhages, atherosclerosis with ruptured plaques, kidney diseases with hematuria, rhabdomyolysis with kidney failure, hemolytic uremic syndromes, diabetic angiopathies, and neonatal retinopathy of prematurity are characteristic disorders for the demonstration of the pathophysiological role of free hemoglobin and heme. Excess free Hb and heme rapidly overwhelm the first line of the endogenous Hb and heme binding homeostatic protective system, the Hb scavenger haptoglobin, and the heme scavenger protein hemopexin and alpha-1-microglobulin. The labile unbound free heme and Hb initiate complex stress reactions, sensitizing cells and tissues towards reactive oxygens species (ROS), provoking cell damage or even cell death.

The second line of the protective system against Hb and heme stress are the intracellular heme oxygenases (HOs) and ferritin. HO enzymes catabolize heme into free ferrous iron, carbon monoxide (CO), and biliverdin (BV) converted to bilirubin (BR) by BV reductases. To eliminate the redox active free iron, cells rapidly express ferritin, an intracellular iron storage protein. The antioxidant character of ferritin depends on its ferroxidase activity and iron sequestering capability [2]. These products of heme catabolism possess a number of physiological functions. BR has been shown to possess remarkable antioxidant effects [3], while CO is an anti-inflammatory and anti-apoptotic gas molecule [4].

Among HOs, HO-1 and HO-2 are extensively characterized [5]. HO-1 is the inducible form, which presents a dramatic intracellular increase of mRNA and protein expressions in response to various environmental stimuli, such as radioactive and ultraviolet irradiation, heavy metals, reactive oxygen species, endotoxin, and several other agents, but most importantly to heme [6,7,8]. Importantly, the dramatic increase in HO-1 mRNA and protein levels does not ultimately correspond to HO-1 activity. In rat models, both Tin(IV)-protoporphyrin (SnPP) and Cobaltic(III)-protoporphyrin (CoPP) dramatically induce HO-1 expression in the liver. However, SnPP completely inhibits HO-1 activity, while CoPP leads to an overall increase in HO-1 catalytic activity [9,10]. The HO-2 enzyme is generally regarded as a constitutively expressed isoform; however, HO-2 expression also changes in response to hypoxia [11]. In addition, studies underline that HO-2 protects neurons against ischemia/reperfusion injury and oxidative damage [12,13].

Among cellular stress reactions, ER stress is one of the best characterized form of stress. The homeostasis of the ER is a finely tuned system; if newly synthesized misfolded or unfolded protein loads exceed the folding capacity of the ER, the pathways of unfolded protein response (UPR) are activated, leading to ER stress. Overwhelming evidence shows that ER stress is involved in a number diverse pathologies, such as diabetes [14], neurodegenerative diseases [15], rheumatic disorders [16], lung disease [17], and atherosclerosis [18]. Since heme is well known as a cell stressor, the hypothesis has to emerge that there must be a close relationship between ER and heme stress.

In the current article, we review the heme–heme oxygenase–ER stress relationship; the major mechanisms of their interactions by which ER stress contributes to the cell and organ damage in diabetes, atherosclerosis, and brain hemorrhage. Since HO-1 presents a unique Janus-faced character in brain pathologies, this issue has received special attention.

## 2. Heme Stress

From an evolutionary perspective, protoporphyrin ring is a unique metal chelator with outstanding properties. Chlorophyll, a magnesium-protoporphyrin, converts light energy to chemical energy and produces organic compounds. Heme, an amphipathic iron-protoporphyrin complex, is one of the most important prosthetic groups on Earth, which serves as an oxygen transporter and participates in various oxido/reductive processes in aerobic and anaerobic cell metabolism. However, heme released from the safe sanctuary area of heme proteins triggers a number of adverse effects. In ’70s and ’80s, several reports revealed the detrimental role of Hb in brain damage [19,20], the heme toxicity in malaria [21], and the potential role of heme in health and disease [22]. Ten years later, we were the first in the literature to be able to generate heme toxicity in cell cultures. We have shown that heme, liberated from heme proteins, is toxic and sensitizes vascular endothelial cells against oxidative stress [2]. Heme, due to its amphipathic nature, shows high affinity towards biological membranes, sensitizing them towards reactive oxygen species (ROS) and leading to the oxidative damage of membrane lipids [23,24], cell lysis [25], genomic [26], and mitochondrial DNA damage [27]. Free heme also triggers protein oxidative modifications [28] and activates the UPR pathways leading ER stress [29]. Moreover, both oxidized Hb and heme are endogenous pro-inflammatory agonists [30,31]. Heme also sensitizes cells to oxidative damage [32] and inflammatory cytokines [33]. Additionally, heme triggers oxidative modifications of lipid particles, such as low-density lipoprotein (LDL), promoting the progression of atherosclerosis [34], which will be discussed later in this review.

Considering the protective effect of HO-1, it is a logical explanation that end-products of heme degradation, BV/BR, and CO are responsible for the beneficial action. There are several experimental conditions where HO-1 provides defense for cells and tissues. Moreover, BR is considered to be an endogenous antioxidant in several clinical conditions. CO and its slow releasing agents (CO releasing molecules, CORMs) have been used as potential medicine, not only in cell culture models, but also in animal studies and clinical investigations. The scientific importance of CO is increasing, since quite a lot of work proves its anti-inflammatory effect.

The nuclear factor-E2-related factor-2 (Nrf2) is activated by diverse environmental stimuli such as oxidative stress, electrophilic, and xenobiotic compounds and plays a pivotal role in coping with oxidative stress [35]. Activation of Nrf2 triggers its dissociation from cytosolic Kelch ECH associating protein 1 (Keap-1) with the subsequent translocation of Nrf2 to the nucleus, where it binds to stress- or antioxidant-response elements (StRE/ARE) encoding a number of genes regulating redox homeostasis, such as HO-1, NAD(P)H:quinone oxidoreductase, glutathione S-transferases, glutamate-cysteine ligase, and glutathione oxidases [36]. On the other hand, HO-1 expression can also be induced by other transcription factors, such as Yin Yang 1 (YY1) [37], activator protein-1 (AP-1) [38], Bach 1 [39], and hypoxia inducible factor 1 [40]. Since HO-1 is abundantly induced by a broad spectrum of endogenous and exogenous stimuli, it is considered as an ideal cytoprotective enzyme.

## 3. Endoplasmic Reticulum Stress

The homeostasis of the endoplasmic reticulum (ER) is a finely tuned system. If newly synthesized misfolded or unfolded protein loads exceed the folding capacity of the ER, the pathways of unfolded protein response (UPR) are activated, leading to ER stress activating pancreatic ER kinase-like ER kinase (PERK), activating transcription factor-6 (ATF6), and inositol-requiring enzyme 1α (IRE1α), all of which are controlled by glucose-regulated protein 78 kDa (Grp78) [41]. PERK directly phosphorylates eukaryotic initiation factor 2α to reduce protein loads of the ER [42] and facilitates the expression of genes involved in the functional UPR, such as activating transcription factor 4 (ATF4), which controls genes involved in amino acid metabolism and redox homeostasis [43]. Severe or unresolved ER stress leads to cell death mediated by the pro-apoptotic transcription factor DNA-damage-inducible transcript 3 (CHOP), which is predominantly induced by the PERK/ATF4 pathway [44]. CHOP is a multifaceted transcription factor with various functions during ER stress. CHOP overexpression triggers apoptosis, whereas CHOP deficient cells are highly resistant to ER stress-induced cell death [45,46]. Furthermore, CHOP plays a key role in lipopolysaccharide-induced inflammation through the induction of caspase-11 [47]. Together, CHOP is an important factor in the pathogenesis of ER stress related diseases and largely determines the fate of cells in response to ER stress [48].

The second major effector protein of ER stress is IRE1α, which splices X-box binding protein (XBP1) mRNA, resulting in spliced XBP1 (XBP1s). XBP1s activates the machineries of endoplasmic reticulum-associated degradation (ERAD), which abolishes unfolded or misfolded proteins [49]. However, IRE1α can also induce cell death by apoptosis signal-regulating kinase (ASK1)-c-Jun amino-terminal kinase (JNK) [50].

Activating transcription factor-6 (ATF6), a transmembrane protein of the ER, activates the expression of ER chaperones, including Grp78, Grp94, protein disulfide isomerase, XBP1, and the components of ERAD [51].

**Figure 1:**
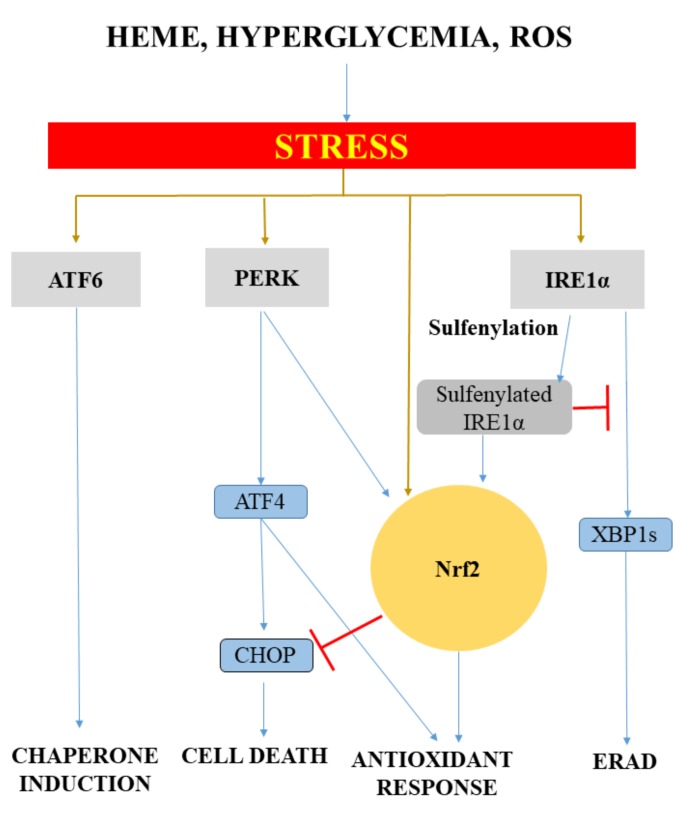
Crosstalk between ER stress and antioxidant response. ER stress in one of the best characterized stress response pathways induced by diverse stress stimuli such as heme, hyperglycemia, and reactive oxygen species (ROS). Activating transcription factor-6 (ATF6) induces ER chaperones, improving protein folding in the ER. Activation of the pancreatic ER kinase-like ER kinase (PERK) arm results in Activating transcription factor-4 (ATF4) expression leading to the activation of antioxidant pathways. In addition, ATF4 also activates the proapoptotic protein DNA-damage-inducible transcript 3 (CHOP) when ER stress is unresolved. Inositol-requiring enzyme 1 (IRE1α) is primarily involved in ER-associated degradation (ERAD) of the damaged proteins. Nuclear factor-E2-related factor-2 (Nrf2), the key regulator of cellular antioxidant response, is strongly connected to the ER stress pathways, since PERK and sulfenylated IRE1α directly activates the Nrf2-mediated stress response. The different consequences of the crosstalk between ER stress and antioxidant response depend on the severity and duration of stress stimuli, as well as the target cell and organ.

Evidence suggests that ER stress pathways interact with Nrf2/HO-1 signaling (Figure 1).

Upon ER stress, PERK directly phosphorylates Nrf2, promoting dissociation from Keap-1 with the subsequent nuclear import of Nrf2 [52]. CO, a by-product of heme degradation by HOs, induces Nrf2 nuclear translocation via PERK activation [53]. In addition, Nrf2-ATF4 dimers bind to the StRE element in the HO-1 promoter, regulating its expression in a cell-specific manner [54,55]. On the other hand, Nrf2 also modulates the expression of certain ATF4-regulated genes such as CHOP, which is negatively regulated by Nrf2 and positively by ATF4 [56]. Another example of the crosstalk between ER residential proteins and antioxidant pathways is IRE1. Reactive oxygen species (ROS) generated by the ER or mitochondria facilitates the cysteine sulfenylation of IRE1 within the kinase loop, which inhibits IRE-1-induced UPR but initiates p38/Nrf2 antioxidant response [57].

Evidence suggests that ER stress pathways interact with Nrf2/HO-1 signaling. Upon ER stress, PERK directly phosphorylates Nrf2, promoting dissociation from Keap-1 with the subsequent nuclear import of Nrf2 [58]. CO, a by-product of heme degradation by HOs, induces Nrf2 nuclear translocation via PERK activation [53]. In addition, Nrf2-ATF4 dimers bind to the StRE element in the HO-1 promoter, regulating its expression in a cell-specific manner [54,55]. On the other hand, Nrf2 also modulates the expression of certain ATF4-regulated genes such as CHOP, which is negatively regulated by Nrf2 and positively by ATF4 [56]. Another example of the crosstalk between ER residential proteins and antioxidant pathways is IRE1. Reactive oxygen species (ROS) generated by the ER or mitochondria facilitates the cysteine sulfenylation of IRE1 within the kinase loop, which inhibits IRE-1-induced UPR but initiates p38/Nrdf2 antioxidant response [57].

## 4. Atherosclerosis

Atherosclerosis is a leading cause of death in developed countries. Labile free heme is one of the many known risk factors for atherosclerosis and contributes to the pathophysiology of this complex disease.

### 4.1. Heme Stress in Atherosclerosis

Hemorrhaged atherosclerotic plaques represent a highly oxidative milieu where invading RBCs are rapidly lysed with subsequent Hb and heme release. Hemorrhage is a frequent complication of plaque development after the rupture of the fibrous cap or intraplaque hemorrhage from the neovasculature budded from vasa plaquorum. Li and co-workers have demonstrated that hemorrhaged atherosclerotic plaques represent a “death zone”, characterized by lipid peroxidation products, which are extremely toxic to the invading cells, including RBCs [59]. In this oxidative scenario, RBCs easily lyse, followed by Hb release and oxidation to form metHb and ferrylHb [60]. MetHb facilitates the oxidant-mediated killing of endothelial cells [61] as well as LDL oxidation via heme release [62,63]. In addition, ferrylHb is a strong proinflammatory agonist that increases endothelial cell permeability and monocyte adhesion [30,64]. These findings corroborate the hypothesis that the oxidation products of free Hb are involved in the progression of atherosclerosis.

Importantly, the lysis of RBCs is not the only fate of erythrocytes during atherosclerosis. ROS can also trigger senescence signals on RBCs, which has been reported in carotid atherosclerosis patients [65]. These senescent state of RBCs possesses remarkable immunomodulatory effects by influencing T cell integrity and function and by affecting the dendritic cell maturation contributing to plaque progression [66].

Recent evidence also shows that heme directly targets the ER of aortic smooth muscle cells, which might be implicated in the pathogenesis of atherosclerosis [29]. This study has demonstrated that heme induces the expression of Grp78 and activates the canonical ER stress pathways, namely PERK, IRE1α/XBP1, and ATF6. In accordance with these findings, higher ER stress marker (Grp78 and CHOP) expression was detected in hemorrhaged atherosclerotic lesions, compared to either atheromas or healthy arteries. Importantly, heme induced ER (HIER) stress is effectively attenuated by the heme scavenger proteins hemopexin and alpha-1-microglobulin. This report highlights that heme directly targets the ER, which might be involved in heme-driven cell damage.

**Figure 2:**
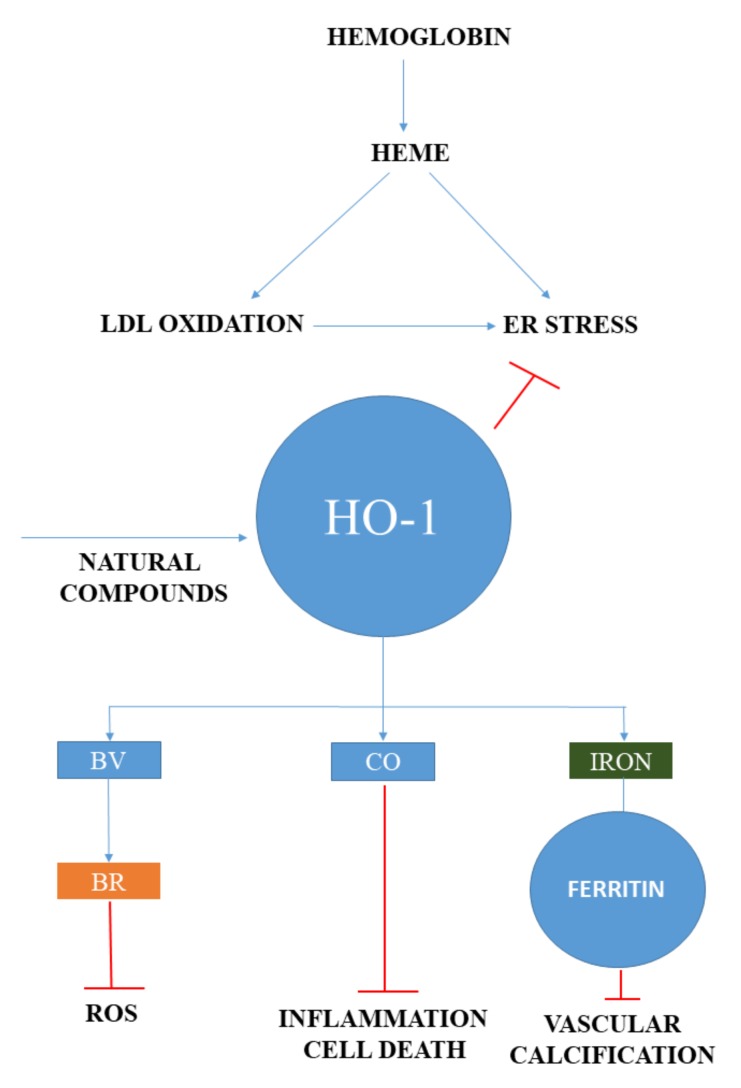
The central role of heme in vascular pathologies. In atherosclerosis, free heme is liberated during hemoglobin oxidation in the vessel wall inducing ER stress and low density lipoprotein (LDL) oxidation. Heme and oxidized LDL induced ER stress is an important factor in the pathogenesis of heme stress, while heme degrading heme oxygenase-1 (HO-1) initiates endogenous protective responses. Biliverdin (BV) is converted into is a natural antioxidant, bilirubin (BR) by biliverdin reductase, carbon monoxide (CO) possesses anti-inflammatory and anti-apoptotic capabilities. Redox-active iron is detoxified and stored by ferritin having ferroxidase activity. In addition, ferritin also mitigates vascular calcification. Natural compounds that induce HO-1 represent a potential therapeutic approach in vascular diseases.

Another mechanism that mediates heme toxicity in atherosclerosis is the oxidative modification of low-density lipoprotein (LDL). Heme, owing to its hydrophobic nature, prefers to associate not only with biological membranes, but also with LDL, representing a physiological mediator of LDL oxidation catalyzed by lipid hydroperoxides that is implicated in the pathogenesis of atherosclerosis [67,68,69]. LDL particles entering the subendothelial area of the arteries are exposed to rapid oxidation, which recruits macrophages and generates foam cells after binding to scavenger receptors on macrophages. Oxidized LDL (oxLDL) is directly cytotoxic to the residential cells of atherosclerotic plaques, such as endothelial cells [70], vascular smooth muscle cells (VSMCs) [71], and macrophages [72]. This oxidative interaction facilitates heme degradation with the subsequent iron release, which further accelerates heme degradation (Figure 2).

### 4.2. HO-1 and Ferritin in Atherosclerosis

LDL oxidized either with heme+hydrogen peroxide or with Cu2+ strongly induces HO-1 in endothelial cells and macrophages [73,74]. In addition, HO-1 is upregulated in the endothelium and foam cells/macrophages of the intima in humans and in apolipoprotein E-deficient (ApoE−/−) mice [74]. The importance of HO-1 in vascular diseases is underlined by the discovery of the first human case of HO-1 deficiency [75]. The lymphoblastoid cell line derived from the HO-1-deficient patient has increased sensitivity to LDL oxidized by heme, compared to control lymphoblast cells with intact HO-1 [76], suggesting that HO-1 is an important factor to mitigate oxLDL-induced oxidative damage.

The pivotal role of HO-1 is also suggested by Cheng et al., who have reported the HO-1 induction reverses plaque progression from a vulnerable to a more stable phenotype [77], which might be mediated by heme catabolism by-products. In addition, plasma HO-1 levels are higher in patients with carotid plaques compared to healthy subjects, which probably indicates a possible protective response against carotid atherosclerosis [78].

It is postulated that pharmacologic HO-1 inducers might be potential candidates for improving atherosclerosis and reducing death in cardiovascular diseases. Mounting evidence suggests that the induction of HO-1 can ameliorate cell injury involved in atherogenesis. Zedoarondiol, an active compound extracted from *Curcuma zedaria*, has been reported to attenuate oxLDL-induced endothelial cell injury and inflammation via upregulation of HO-1 by Nrf2 [79]. Atractylenolide I (AO-I), isolated from *Atractylodes macrocephala*, inhibits ox-LDL-induced VSMCs migration and inflammatory responses partly in an HO-1-dependent manner [80].

### 4.3. BR and CO in Atherosclerosis

BR inhibits monocyte migration across the activated endothelial cells, by scavenging ROS and affecting adhesion molecule signaling, and prevents plaque formation in LDL receptor-deficient mice [81]. In addition, decreased serum BR is associated with the severity of atherosclerosis [82] and low serum levels of indirect BR is an independent predictor of subclinical atherosclerosis [83]. These are in good agreement with a study presenting that chronic moderate hyperbilirubinemia in Gilbert syndrome prevents the development of ischemic heart disease [84]. These findings show that a low serum BR level might be associated with increased risk for atherosclerosis.

CO, due its anti-inflammatory and anti-apoptotic properties, might be a promising candidate to mitigate inflammation and apoptotic cell death in vascular diseases. This hypothesis is supported by Soni et al., who have shown that exogenous CO mitigates doxorubicin-induced cardiotoxicity [85]. CORM-3 improves structural and functional cardiac recovery after myocardial injury [86]. In addition, CORM-2 reduces endothelial cell apoptosis triggered by ox-LDL [87].

Whereas CO might be beneficial in doxorubicin-induced damage, a recent report has shown that exogenous CO may disturb lipid metabolism in macrophages promoting foam cell formation, which is a hallmark of atherosclerosis [88]. To provide a more comprehensive understanding of the potential therapeutic application of CO releasing agent in vascular diseases, the safe therapeutic range of CORMs should be determined because of the toxicity of CO.

Ferritin, which is abundantly induced during heme catabolism by HOs, plays a pivotal role in the detoxification of redox-active iron releases by HOs. Induction of ferritin is also dependent upon the microenvironment within the vessels, because ferritin expression is also regulated by inflammation [89]. Interestingly, ferritin is more than an iron storage protein. Ferritin has been reported to inhibit the calcification of both aortic smooth muscle cells [90] and valvular interstitial cells [91]. Moreover, the pharmacological induction of ferritin also prevents smooth muscle cell calcification [92]. This raises an interesting hypothesis that cardiovascular complications might be mitigated not only by HO but also by ferritin, the other important component of the HO-ferritin system.

Protection against heme and ROS-mediated toxicity by HO-1 is important to maintain vascular homeostasis. Therefore, using pharmacologic approaches, the induction of HO-1 might be beneficial in atherosclerosis.

### 4.4. ER Stress in Atherosclerosis

Numerous reports have shown that ER stress is involved in the pathogenesis of vascular diseases. Elevated intracellular ox-LDL and 7-ketocholesterol provoke ER stress, characterized by a marked CHOP expression with subsequent apoptosis in macrophages and endothelial cells [93,94,95]. Moreover, CHOP expression is also high in unstable atherosclerotic plaques, which is likely to contribute to plaque vulnerability [94]. Additionally, ER stress also promotes foam cell formation in response to ox-LDL [96]. Thus, based on these findings, it is postulated that by mitigating ER stress, atherosclerosis might be resolved or, at least, its progression can be slowed down. In fact, recent studies corroborate this hypothesis. Lipid-mediated toxicity of macrophages is reduced by the chemical chaperone 4-phenylbuturic-acid (4-PBA) [97]. A recent study revealed that another ER stress inhibitor, tauroursodeoxycholic acid (TUDCA), ameliorates atherosclerotic lesions and systemic lipid levels in ApoE−/− mice on a high-fat diet [98].

Even though ER stress and oxidative stress coexist in many pathologies [99], the link between them has not been clearly elucidated. However, it is postulated that Nrf2/HO-1 and ER stress pathways interact and oxidative stress-triggered ER stress might be implicated in atherosclerosis. This theory is supported by Mozzini et al., who showed that GRP78/CHOP expression is increased while Nrf2/HO-1 is decreased in coronary artery disease patients [100]. The authors suggest that phospholipid 1-palmitoyl-2-arachidonyl-sn-glycero-3-phosphorylcholine (oxPAPC), which presumably derives from oxidized LDL, negatively regulates Nrf2/HO-1 expression with a parallel ER stress-inducing effect. In another study, equol, a specific metabolic product of daidzein in soybean, reduces atherosclerotic lesions in ApoE−/− mice fed with high-fat diet via attenuating ER stress in an Nrf2-dependent manner [101].

Isorhamnetin (Iso), a flavonoid compound extracted from *Hippophae rhamnoides L.*, inhibits ox-LDL-induced macrophage injuries by decreasing ROS levels and protecting against ox-LDL-induced apoptosis [102]. In addition, Iso also reduced atherosclerotic plaque size and macrophage apoptosis in ApoE−/− mice. These in vitro and in vivo effects of Iso are mediated by PI3K/AKT activation and HO-1 induction. Interestingly, a recent report shows that Iso also inhibits ER stress, but the possible interplay between this effect and the HO-1-inducing effect of Iso needs further investigation [103].

These results highlight that pharmacologic induction of Nrf2/HO-1 provides a potent antioxidant defense mechanism, which might mitigate the progression of atherosclerosis, at least partly, by attenuating ER stress. These results highlight that little is known about the crosstalk between HO-1 and ER stress in vascular diseases. Further studies may reveal the potential link between antioxidant and ER stress responses and identify molecular targets that govern the pathophysiological events at the cellular level in atherosclerosis.

## 5. Brain

**Figure 3:**
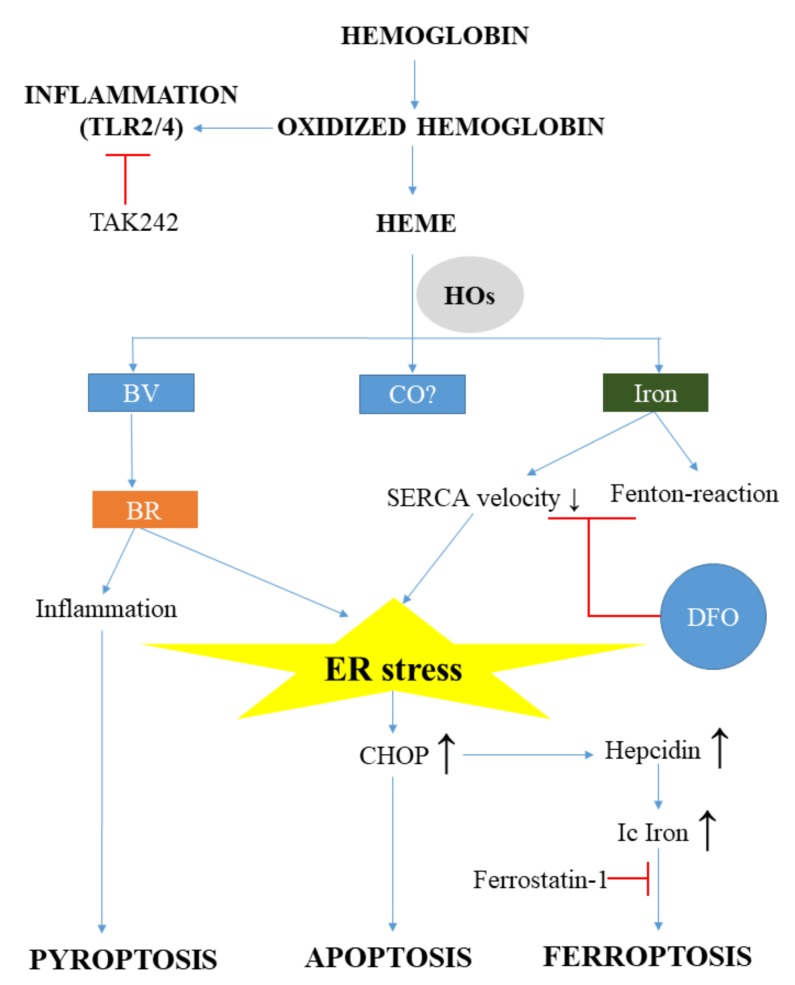
Free hemoglobin and heme participates in the pathophysiology of brain damage. During brain hemorrhage, massive amounts of free hemoglobin and heme are released, resulting in inflammation, heme-, and ER stress. Oxidized hemoglobin directly triggers inflammation via toll-like receptor 2 and 4 (TLR2, TLR4). Heme oxygenases (HOs) in the brain are Janus-faced and might induce both protective and adverse effects. Although bilirubin (BR) is a natural antioxidant, it can induce inflammation, pyroptosis, or even apoptosis through ER stress in the brain. Heme-derived iron also triggers ER stress. Elevated intracellular iron (Ic Iron) derives from HO activity and the disturbed cellular iron metabolism through hepcidin induction, leading to ferroptosis. (SERCA: sarco/endoplasmic reticulum Ca2+-ATPase; DFO: desferrioxamine; CHOP: C/EBP homologous protein; CO: carbon monoxide; BV: biliverdin; TAK: Ethyl-(6R)-6-(N-(2-chloro-4-fluorophenyl)sulfamoyl)cyclohex-1-ene-1-carboxylate; TLR: Toll-like receptor

Lesions of the central nervous systems are not only based on primer brain pathologies, but also on secondary events. Analyzing the nature of brain lesions, there are several ones where the presence of heme and ER stress is obvious and well documented. The intracerebral hemorrhage (ICH) is a classical type of brain vascular disease gaining importance presently, as the ageing of the population and the use of anti-coagulants rises. The initiation step of ICH is the accumulation of blood within the brain tissue with the subsequent lysis of RBCs resulting in massive Hb and heme release. The breakdown of heme by HOs yields a large amount of redox-active iron, (CO) and BV, which is converted to BR. Hard evidence show that Hb, heme, and heme catabolism by-products are all involved in the pathogenesis of ICH (Figure 3).

MetHb is an endogenous ligand of toll-like receptor 2 (TLR2) and toll-like receptor 4 (TLR4) that induces pro-inflammatory cytokine and tumor necrosis factor α (TNF-α) production after hemorrhage [104,105]. Heme is also pro-inflammatory for many cell types, such as macrophages and microglia and activates TLR4-mediated inflammatory injury in ICH, which is markedly reduced by knocking down TLR4 [106,107]. In addition, heme binds to TLR2 and severely triggers blood–brain barrier damage in astrocytes in a TLR2 dependent manner [108]. These findings suggest that the modulation of TLR signaling might be a promising therapeutic target in hemorrhagic brain injury. This hypothesis has been tested by Wang et al., who showed that the TLR4 antagonist Ethyl-(6 R)-6-(N-(2-chloro-4-fluorophenyl)sulfamoyl)cyclohex-1-ene-1-carboxylate (TAK242) reduces inflammatory injury and neurological deficits in mice after hemorrhage [109]. Although this is an interesting approach, further research is needed in this field to explore the effect of TLR4 antagonists in immunohistochemistry (IHC).

### 5.1. ER Stress in Brain Injury After Hemorrhage

ER stress plays a significant role in brain injury after hemorrhage. Hemorrhage induced ER stress primarily targets neurons and the protein kinase R (PKR)-like endoplasmic reticulum kinase (PERK)/CHOP pathway might have predominant role in neuronal cell death. Several lines of evidence support this theory. In a rat model of cerebral ischemia/reperfusion injury, silencing of CHOP by the lentivirus-mediated transfer of short hairpin RNA mitigated inflammatory and apoptotic reactions in neuronal cells, suggesting that CHOP silencing is neuroprotective [110]. PERK signaling is activated following subarachnoid hemorrhage (SAH) and PERK inhibitor GSK2606414 reduces neuronal cell death via Akt activation [111]. This has also been corroborated by another study, which shows that the PERK pathway is involved in neuronal loss and apoptosis following ICH, which is attenuated by PERK inhibition [112]. Overall, these studies indicate that the PERK pathway plays an important role in brain injury after hemorrhage and PERK inhibitors might be potential candidates for mitigating ER stress-induced cell death and brain damage after SAH.

### 5.2. HO-1 and HO-2 in the Brain

The role of HO-1 in brain hemorrhage is highly controversial. In a murine model of SAH, Schallner and co-workers have demonstrated that inducible HO-1 in microglia is necessary to reduce neuronal cell death and to clear cerebral blood [113]. In contrast, the injury volume in HO-1 knockout (HO-1^−/−^) mice is significantly smaller 24 and 72 h after the injury, compared to wildtype mice [114]. In addition, pharmacological inhibition of HO-1 with tin-mesoporphyrin also protects against neuronal loss in the rabbit model of ICH [115]. Others suggest that the effect of HO-1 is time dependent. In a collagenase-induced ICH murine model, Zhang and co-workers have suggested that HO-1 induction is Janus-faced. During the early-phase of ICH (day 1–3), HO-1 increases brain edema, white matter and neuronal damage, and elevates inflammation and iron deposition, but in the late-phase (day 28), HO-1 increases hematoma absorption and recovery of neurologic functions [116]. On the other hand, Wang et al. suggest that HO-1 is protective in the early-mid phase, but in the late phase, it might be toxic [117].

The role of HO-2 in brain hemorrhage is similarly controversial. In HO-2 knockout (−/−) mice, HO-2 deficiency worsens neurotoxicity mediated by stroma-free hemoglobin [118]. Others using HO-2 knockout mice support that HO-2 has a critical protective effect in IHC [12]. On the contrary, Chen-Roetling and co-workers suggest that, in the blood injection ICH model, neuronal survival is markedly increased in HO-2 knockout mice compared to wild-type mice [119].

Despite many years of research on the subject of how HO-1 and HO-2 may influence the pathomechanism and recovery after ICH, results are controversial, which might explain the diverse ICH models. Furthermore, not only heme but also its breakdown products, namely iron and BR, catalyze adverse reactions in the brain and contribute to brain damage after hemorrhage.

### 5.3. Iron Injury in the Brain

Free heme is rapidly taken up by microglia and invading macrophages, and within a few hours after hemorrhage it is catabolized into redox-active iron, CO, BV, and BR. One feasible mechanism of heme toxicity may be the release of free redox-active iron by HOs. Activation of HO-1 in response to heme is closely coupled with the induction of ferritin, an intracellular iron-storage protein with potent ferroxidase activity. It is likely that cells are not able to accumulate ferritin continually and this iron-scavenging homeostatic mechanism may fail after massive hemorrhage, resulting in a robust redox-active iron burden. Redox-active iron strongly promotes free radical formation by the Fenton-reaction, triggering oxidative damage in biological systems [120]. Importantly, free redox-active iron also promotes ER stress and this iron-induced ER stress is likely to contribute Hb/heme toxicity in the brain. In vitro and in vivo models support this tenable hypothesis, i.e., ER stress is a possible etiological factor in brain hemorrhage.

Ferrous iron induces both lipid-mediated and lipid-peroxidation independent changes in the ER membrane-associated proteins in brain [121]. Free radicals generated by redox-active iron decrease the velocity of sarco/endoplasmic reticulum Ca^2+^ ATPase (SERCA) [122]. This resembles the inhibitory mechanism of thapsigargin, a well-characterized ER stress inductor, which also inhibits SERCA. Therefore, one reasonable assumption might be that redox-active iron-induced cell damage is mediated via ER stress and factors regulating iron metabolism may influence brain damage. One of these factors might be hepcidin, which prevents iron export from cells by breaking down the iron transporter ferroportin. Remarkably, ER stress upregulates hepcidin expression [123]. This connection has been revealed in the brain by a recent study. Zhao and co-workers have shown the ER stress markers Grp78 and CHOP are induced in rats after subarachnoid hemorrhage and neuronal death is mediated by hepcidin [124]. Disturbed iron homeostasis might lead to an iron-dependent form of cell death, ferroptosis [125]. CHOP increases hepcidin levels and iron content in the brain, which localizes to the nuclei of neurons. Importantly, knocking-down CHOP improves neurological functions. These observations support the hypothesis that brain injury after hemorrhage is, at least partly, mediated by ER stress.

Previous studies have demonstrated that desferrioxamine (DFO) and other iron chelators are neuroprotective in brain hemorrhage [126,127]. Interestingly, a recent report makes this iron toxicity model more complex. LeBlanc and co-workers have shown in trans-well experiments with microglial and hippocampal neuronal cells that DFO markedly reduces RBC-mediated neuronal cell death, but this protective effect is highly dependent on microglial HO-1 [128].

### 5.4. BR Toxicity in Brain

BR is an important endogenous antioxidant [129,130]. However, BR neurotoxicity is well-founded, especially in newborns, and necessitates phototherapy [131,132] and, in extreme cases, exchange transfusion [133]. In addition, BR levels and toxicity should be more closely monitored in newborns with hemolytic disease [134]. Interestingly, inhibiting HO-1 catalytic activity by SnPP markedly mitigates hyperbilirubinemia in preterm newborns [135].

After hemorrhage, massive amounts of unconjugated bilirubin (UCB) might be produced by HOs during heme degradation. In hepatocytes, UCB is conjugated to glucuronides in the ER, which indicates that UCB has a high affinity towards the ER [136].

UCB has been reported to induce ER stress, inflammation, and even apoptosis in SH-SY5Y neuronal cells, while knocking down of CHOP or attenuating ER stress by 4-PBA markedly reduced cell death [137]. A current study supports this observation. Schiavon and co-workers showed that BR induces ER stress and inflammation, both in vitro and in vivo, and raised the auditory threshold together with behavioral impairment in the murine model [138]. Another form of UCB induced cell death, that is presumably pyroptosis, a highly inflammatory form of programmed cell death, has been revealed in rat cortical astrocytes [139].

These findings corroborate the fact that BR neurotoxicity and BR-induced ER stress might be involved in brain damage after hemorrhage.

### 5.5. CO in the Brain

Bioactivity of CO as the potential anti-inflammatory, anti-apoptotic, and antioxidant substance has been reviewed extensively in the past few years [4]. Based on these beneficial properties of CO, carbon monoxide-releasing molecules (CORMs) should be ideal candidates for reducing inflammation and cell death after brain hemorrhage. However, relevant data in this field are scarce.

CORM-3, a water-soluble CO-releasing molecule, promotes either neuroprotection or neuroinflammation, depending on the administration time in collagenase-induced ICH model [140]. It is neuroprotective when administered 5 min before the IHC and in the subacute phase, 3 days after the hemorrhage, but it aggravates brain injury and tumor necrosis factor-α production administered in the acute phase, 3 h after the IHC. Further research is needed to explore the possible beneficial effect of CORMs to mitigate brain injury after hemorrhage.

Overall, despite extensive research, the role of HOs in protecting brain homeostasis after hemorrhage remains elusive. However, it is likely that ER stress is deeply involved in heme toxicity in the brain and CHOP plays an important role in many aspects of this toxicity by regulating iron and BR induced cell damage.

## 6. Diabetes Mellitus

The long-term complications of diabetes mellitus, micro- and macro-vascular pathologies, largely influence quality of life and mortality. Diabetes poses a significant threat and economic burden, especially in developed countries. In this review, we will focus on the role of HO-1 in diabetic cardiovascular complications.

### 6.1. Diabetic Cardiovascular Complications 

Whereas oxidative stress is significantly increased in diabetic patients, the total antioxidant status is decreased [141]. Hyperglycemia promotes oxidative stress by free radical generation and suppresses antioxidant defense [141,142]. Diabetes, together with hyperglycemia-induced oxidative stress, is a major risk factor for accelerated atherosclerosis [143]. Hyperglycemia lowers HO-1 activity and increases superoxide production in the vasculature, which is mitigated by CoPP [144,145]. In addition, both upregulation of HO-1 or production of CO with CORM-3 have beneficial effects on vascular relaxation in rats [144,146]. Interestingly, obesity also decreases HO-1 levels in both male and female rats, compared to lean animals, and the induction of HO-1 with CoPP reduces blood pressure and inflammatory cytokine levels [147].

Since the endogenous anti-oxidant response is impaired in diabetes, it is rational that resolving imbalances in the antioxidant system might restore redox homeostasis. This has been seen in the case of hydrogen sulfide (H_2_S), which reduces aortic atherosclerotic plaque formation by detoxifying superoxide [148]. This protective effect of H_2_S is mediated by HO-1 activation via the Keap-1/Nrf2 system. In addition, HO-1 induction by hemin restores the cardioprotective effect of ischemic preconditioning in diabetic rat heart [149]. Moreover, activation of HO-1 with hemin mitigates renal damage in streptozotocin (STZ)-induced diabetic nephropathy in rats, by reducing inflammation and apoptosis, and improves antioxidant response [150].

Another interesting therapeutic approach might be the epigenetic regulation of HO-1 expression. MicroRNAs (miRNAs) are small noncoding RNAs involved in the post-transcriptional regulation of protein expression. An interesting example by which HO-1 is regulated by miRNAs is microRNA-92a (miR-92a). The miR-92a expression is increased in diabetic endothelial cells. A recent report shows that miR92a negatively influences HO-1 expression [151] and the inhibition of miR-92a elevates HO-1 expression, mitigates oxidative stress, and improves endothelial function in diabetic mice. The importance of miRNAs in HO-1 regulation in diabetes is emphasized by the fact that other miRNAs, such as miR-218, induce cell death in podocytes by downregulation of HO-1 expression [152]. Together, an interesting epigenetic therapeutic approach would be the targeted induction of HO-1 in endothelial cells to prevent the diabetic dysfunction of these cells.

Data on the role of HO-2 in diabetes are scarce. It has been reported that HO-2 deficiency increases superoxide production and contributes to renal dysfunction in a diabetic rat model [153]. Very little is currently known about the protective role of HO-2 to cope with oxidative stress in diabetes, which necessitates further research in this field.

### 6.2. HO-1 Byproducts in Diabetes

**Figure 4:**
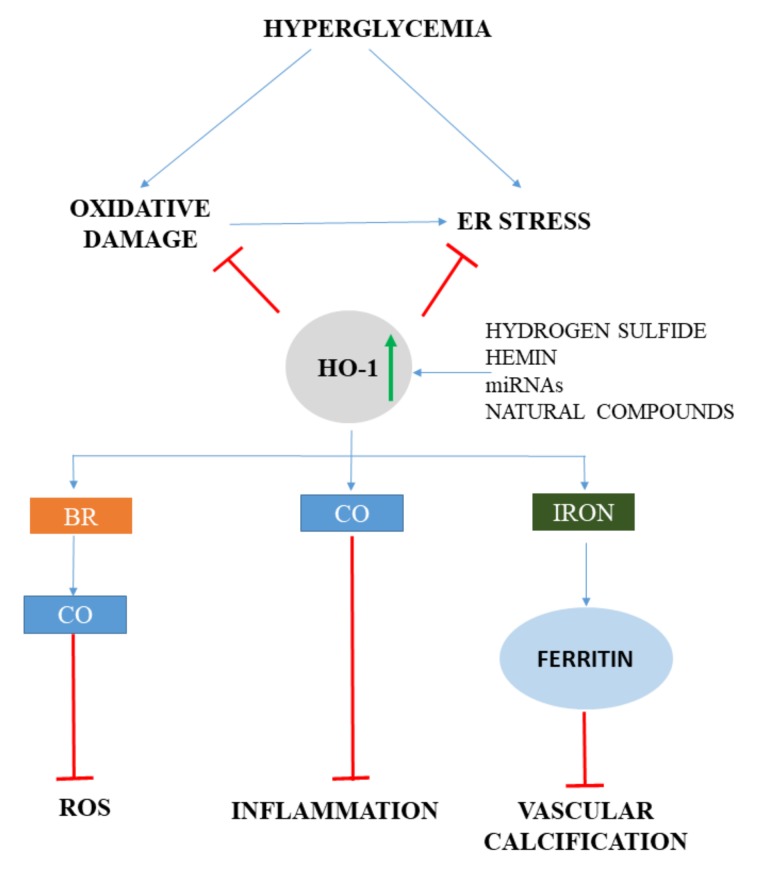
HO-1 mitigates oxidative and ER stress in diabetes. Hyperglycemia induces oxidative stress as well as ER stress, which is attenuated by HO-1. Bilirubin (BR), carbon monoxide (CO), and ferritin are all involved in decreasing diabetic vascular complications by reducing oxidative damage, inflammation, and vascular calcification. Pharmacologic and natural HO-1 inducers are potential candidates to reduce diabetic vascular complications. (HO: heme oxygenase; ROS: reactive oxygen species; miRNA: micro ribonucleic acid)

BR provides a potent antioxidant defense mechanism in response to oxidative stress, suggesting that BR might mitigate ROS-induced cell- and tissue damage in diabetes. Elevated glucose concentrations trigger ROS generation and induce HO-1 expression both in vivo and in vitro. Inhibition of HO-1 activity exacerbates high glucose-induced oxidative cell injury, which is markedly attenuated by BR [154]. Diabetes and hyperglycemia induce oxidative stress, leading to endothelial dysfunction, which is a hallmark of diabetic vascular complications. It has been demonstrated that BR is implicated in HO-1-mediated restoration of impaired endothelial function in diabetic mice [155]. In addition, lower serum BR, together with the neutrophil-lymphocyte ratio, are independent predictors of subclinical atherosclerosis in prediabetes [156]. In murine model, BR increased insulin sensitivity by reducing ER stress and inflammation [157]. This suggests that BR is more than a potent antioxidant and it might be useful as an insulin sensitizer in type 2 diabetes by reducing hyperglycemia-induced ER stress and inflammation (Figure 4).

CO possesses remarkable anti-apoptotic and anti-inflammatory properties. Carbon monoxide-releasing molecules (CORMs) are valid and safe alternatives to the CO gas-based therapies and exert protective effects in diabetes-induced inflammation. In a mouse model of STZ-induced diabetes, CORM-3 markedly reduced hyperglycemia-induced inflammation by decreasing IL-1β production [158]. Others have demonstrated that hemin, as well as BR and the CO donor CORM-2, reduces hyperglycemia and improves the abnormality of endothelium-dependent vascular relaxation in STZ-induced diabetic rats [159]. These studies suggest that CORMs might have beneficial effects to reduce inflammation in diabetes as supportive therapy.

Coronary, carotid, and aortic valve calcification are frequent complications of diabetic patients [160,161], which might be mitigated by ferritin as discussed above.

Overall, a body of evidence suggests that HO-1, ferritin, BR, and CO are all capable of reducing oxidative stress and attenuating ROS-drive cell and tissue damage.

### 6.3. ER Stress in Diabetes

Mounting evidence suggests that ER stress is involved in the pathogenesis of diabetes and plays a pivotal role in diabetic complications, such as neuropathy [162], diabetic nephropathy [163], diabetic retinopathy [164], and cardiomyopathy [165,166]. HO-1 and ER stress pathways interact in diabetes (Figure 4). In type 2 diabetic patients, the ER stress marker expression with concomitant oxidative stress is significantly higher, while anti-inflammatory Inhibitor of κB-α (IκB-α) and Nrf2/HO-1 are significantly lower compared to healthy controls [167]. Parallel with that, prolonged hyperglycemia induces NFκB without a Nrf2 response. This demonstrates that hyperglycemia might trigger ER stress, inflammation, and oxidative stress without Nrf2 activation. Hyperglycemia also induces ER stress, inflammation, and apoptosis in endothelial cells, which is attenuated either by inducing HO-1 with Cobalt (III)-Protoporphyrin IX chloride (CoPPIX) or by reducing ER stress by 4-PBA [168]. In addition, both CoPPIX and 4-PBA reduce the angiogenic capacity of human umbilical vein endothelial cells (HUVECs) and increases vascular endothelial growth factor-A (VEGF-A) expression. This observation underlines that ER stress plays a role in diabetes-induced endothelial dysfunction and impaired angiogenesis and HO-1 induction might be a protective stratagem to mitigate the adverse effects of ER stress in diabetic cardiovascular complications.

Natural compounds that activate Nrf2/HO-1 pathways are promising therapeutics to reduce ER stress, as well as oxidative stress, in diabetes. Tangluoning, a traditional Chinese medicine, has been reported to activate the PERK/Nrf2 pathway upregulating antioxidant responsive element (ARE) elements including HO-1, thereby attenuating diabetic peripheral neuropathy and CHOP-mediated apoptosis [169]. Grape seed proanthocyanidins (GSP) mitigate early diabetic peripheral neuropathy by modulating endoplasmic reticulum stress and preventing calcium overload [170]. Interestingly, GSPs are effective activators of Nrf2 and HO-1, which suggests the protective effect of GSPs in diabetic neuropathy.

Chrysin, a naturally occurring flavonoid found in various herbs, reduces high glucose-induced ER stress in retinal pigment epithelial cells and might be beneficial to attenuate diabetes-associated visual cycle impairment implicated in diabetic retinopathy [171]. Another study revealed that chrysin is also a potent inductor of HO-1 [172].

Arctigenin (ATG), a lignan extract from *Fructus arctii*, has renoprotective effects on diabetes-related renal injury by inhibiting ER stress and apoptosis [173]. It is not surprising that ATG also induces HO-1 [174].

These studies possibly reveal that the crosstalk between ER stress and Nrf2/HO-1 mediates the beneficial effects of these natural compounds in hyperglycemia-induced complications. It still remains a remarkable question whether the effects of these compounds are attributed or not to HO-1 induction. Inhibition of HO-1 activity or by knocking down/out HO-1 might provide a mechanistic model by which the exact role of HO-1 can be revealed in ER stress in diabetes.

### 6.4. Brain and Diabetes

Another serious complication of diabetes is the diabetes-associated cognitive decline. In an STZ-induced diabetic mouse model, increased ER stress, JNK activation, and autophagy have been observed in hippocampal neurons [175]. Importantly, 4-PBA attenuates neuronal cell death in mice, while autophagy inhibitor bafilomycin A1 increases cell death in vitro.

A recent report has revealed that HO-1 expression in response to hyperglycemia might also have adverse effects [176]. Hyperglycemia induces HO-1 expression in rat astrocytes in vitro and conditioned medium from high glucose-treated astrocytes triggers cell death in neuronal cells. Interestingly, hemoglobin treatment, as a CO scavenger, prevents neuronal cell death provoked with high glucose conditioned medium, suggesting that HO-1/CO activation in astrocytes provokes neuronal cell death. These findings raise the hypothesis that HO-derived CO might play a negative role in diabetes-induced complications in the brain.

## 7. Discussion

Crosstalk between oxidative stress and ER stress markedly contributes to the pathogenesis of vascular diseases, as well as diverse pathologies associated with heme stress. Therefore, ER stress inhibitors might be ideal candidates to ameliorate the adverse pathophysiological consequences of these diseases when the endogenous homeostatic defense is overwhelmed. HO-1 is implicated in numerous cellular protective pathways and might be involved in managing ER stress due to its antioxidant, anti-inflammatory, and anti-apoptotic effects, mediated directly by HO-1 or by heme catabolism end-products, i.e., CO, bilirubin, and iron. However, HO-1 activity might pose a threat to the homeostasis of cells by releasing vast amounts of these agents when free heme is present. HO-1 induction by natural compounds is beneficial, in many aspects, by reducing ER stress and oxidative damage; however, the exact role of HO-1 in mitigating ER stress remains to be elucidated.
